# Novel Cold Crucible Ultrasonic Atomization Powder Production Method for 3D Printing

**DOI:** 10.3390/ma14102541

**Published:** 2021-05-13

**Authors:** Łukasz Żrodowski, Rafał Wróblewski, Tomasz Choma, Bartosz Morończyk, Mateusz Ostrysz, Marcin Leonowicz, Wojciech Łacisz, Piotr Błyskun, Jan S. Wróbel, Grzegorz Cieślak, Bartłomiej Wysocki, Cezary Żrodowski, Karolina Pomian

**Affiliations:** 1 Faculty of Materials Science and Engineering, Warsaw University of Technology, Woloska141 St., 02-507 Warsaw, Poland; tomasz.choma@amazemet.com (T.C.); bartosz.moronczyk.dokt@pw.edu.pl (B.M.); marcin.leonowicz@pw.edu.pl (M.L.); piotr.blyskun@pw.edu.pl (P.B.); jan.wrobel@pw.edu.pl (J.S.W.); grzegorz.cieslak@pw.edu.pl (G.C.); kp.pomian@wp.pl (K.P.); 2 AMAZEMET Sp. z o.o. [Ltd], Al. Jana Pawła II 27, 00-867 Warsaw, Poland; mateusz.ostrysz@amazemet.com (M.O.); wojciech.lacisz@amazemet.com (W.Ł.); 3 Center of Digital Science and Technology, Cardinal Stefan Wyszynski University in Warsaw, Woycickiego 1/3, 01-938 Warsaw, Poland; b.wysocki@uksw.edu.pl; 4 MaterialsCare LCC, Zwierzyniecka 10/1, 15-333 Bialystok, Poland; 5 Faculty of Ocean Engineering and Ship Technology, Gdansk University of Technology, 80-233 Gdansk, Poland; cezary.zrodowski@pg.edu.pl

**Keywords:** ultrasonic, powder atomization, cold crucible, additive manufacturing, powder metallurgy, recycling

## Abstract

A new powder production method has been developed to speed up the search for novel alloys for additive manufacturing. The technique involves an ultrasonically agitated cold crucible installed at the top of a 20 kHz ultrasonic sonotrode. The material is melted with an electric arc and undergoes pulverization with standing wave vibrations. Several different alloys in various forms, including noble and metallic glass alloys, were chosen to test the process. The atomized particles showed exceptional sphericity, while powder output suitable for additive manufacturing reached up to 60%. The AMZ4 metallic glass powder remained amorphous below the 50 μm fraction, while tungsten addition led to crystallization in each fraction. Minor contamination and high Mn and Zn evaporation, especially in the finest particles, was observed in atomized powders. The innovative ultrasonic atomization method appears as a promising tool for material scientists to develop powders with tailored chemical composition, size and structure.

## 1. Introduction

Additive manufacturing (AM) techniques such as Laser Powder Bed Fusion (LPBF) or electron beam melting (EBM) required powders with exceptional flowability [1] to ensure process repeatability and high density [2] of printed parts. Typically, such powders are produced via gas atomization [3]; although, due to the industrial scale of atomizers, the search for new alloys fine-tuned for AM is expensive and limits the number of compositions able to be tested [4]. Such limitation is particularly troublesome during chemical optimization of precious metal powders such as gold [5] and highly reactive [6] or radioactive materials [7]. The main limitation of gas and centrifugal atomization is associated with the high speed of pulverized particles during the atomization process, which requires a large sized atomization tower [8,9]. The method based on radio-frequency plasma spheroidization requires powder as a raw feedstock; [10] thus, its application is mostly limited to brittle materials such as refractory metals and titanium hydrides.

Ultrasonic atomization is one of the least studied methods of melt atomization. Instead of a high-velocity gas or centrifugal force, the acoustic wave breaks the internal forces of molten metal [11]. Vibrations can be introduced directly by means of a sonotrode (ultrasonic tool) [12] or through a vibrating medium [13]. This method has been widely used for solders [14], the production of aluminum [15], zinc [16] and magnesium alloys [17] or alloys with a higher melting point such as steel [18] and titanium [19]. As shown by Y. Goto et al. [19], the working time and the amount of atomized powder is limited by the frequency shift of the heated vibrating elements. The novel approach is based on continuously cooled ultrasonic tools operating under constant heat flux conditions and is designed for a laboratory quantity of spherical powder for AM.

In this article, we present a new, universal method of ultrasonic powder atomization with the use of various forms of feed material. The atomized powder was characterized and compared with other production methods. The materials examined in the paper were selected in such a way that they form two groups: (1) well-known metal and commercial alloys such as Ti, 304 steel and Au-based alloy for method validation; and (2) bulk metallic glasses and high-entropy alloys for testing their suitability for novel materials development.

Bulk metallic glasses (BMGs) are non-crystalline metallic alloys, which can be produced at low cooling rates in various alloy systems. Thus, it is possible to produce BMGs with a thickness of several centimeters. BMGs exhibit useful engineering properties such as high corrosion resistance and mechanical strength; they are important multicomponent alloys with innovative microstructures and unique properties that make them promising in a number of industries. However, some obstacles to the production of BMG by conventional techniques have been identified due to their inherent requirements [20,21]. With the advent of metal additive manufacturing, new possibilities have arisen for the production of geometrically complex BMGs with tailored microstructures, theoretically anywhere in the sample, which cannot be achieved with conventional fabrication techniques [22].

High-entropy alloys (HEAs) are multicomponent systems incorporating four or more elements in similar concentrations, unlike existing commercial alloys. The conventional concept behind HEAs is that the high configurational entropy inhibits the formation of brittle intermetallic phases in favor of the disordered fcc or bcc phase. Due to their microstructural simplicity that requires unsophisticated thermal treatment, they are considered as promising candidates for AM technology. It has been proven that HEAs can be produced by AM and that they have very good mechanical properties [23,24]. The Fe–Cr–Mn–Ni alloys were selected for atomization because of their excellent irradiation properties, making them potential candidates for fission and fusion applications [25].

## 2. Materials and Methods

**Figure 1:**
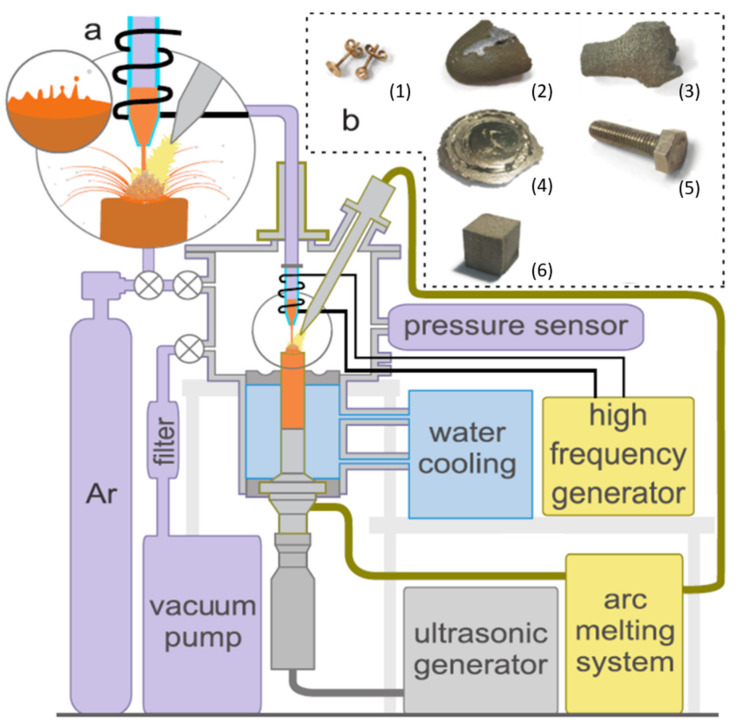
Process diagram and experimental setup (**a**), material feedstock (**b**): (1) Au-based alloy earrings, (2) AMZ4 (Heraeus GmbH) Zr-based metallic glass, (3) slabs of Ni–Mn–Ga magnetocaloric alloy, (4) forged equiatomic Fe–Cr–Mn–Ni high entropy alloy, (5) AISI 304 steel as a M10 bolt, (6) Grade 2 titanium porous implant.

As shown in Figure 1a, the system consists of an ultrasonic stack (1), an arc melting system (2) and a vacuum chamber (3). The ultrasonic unit consists of an ultrasonic transducer, a titanium 1:2 amplitude booster and an Ampcoloy® 972 sonotrode (Amazemet Ltd., Warsaw, Poland). The ultrasonic transducer has a nominal frequency of 20 kHz, maximum power of 5 kW and an operating frequency of ± 500 Hz. The amplitude booster serves as an electrical grounding. The sonotrode is a critical part of the system, being both a cold crucible and an ultrasonic tool. The arc system provides up to 400 A direct current. The system operates in high purity argon after purging several times under a vacuum of 10^−2^ bar.

To demonstrate the operation of the system, several different materials were selected to be atomized: AMZ4 (Heraeus GmbH, Hanau, Germany) Zr-based metallic glass and AMZ4 alloy with 20% tungsten, AISI 304 steel as the M10 bolt, a Grade 2 titanium porous implant, slabs of Ni–Mn–Ga magnetocaloric alloy, forged equiatomic Fe–Cr–Mn–Ni high-entropy alloy and Au-based alloy earrings. To atomize the materials, 300 A current and 35 µm amplitude parameters were chosen for the sonotrode. Such parameters constitute maximum achievable values for the system and optimization of the process is beyond the scope for the paper. The materials underwent a sieve analysis to compare the particle size distribution in various alloys. AMZ4, AMZ4 + W, Ni–Mn–Ga and Fe–Cr–Mn–Ni powders were subjected to X-ray phase analysis with the Rigaku Miniflex II XRD. The chemical composition of feedstock and atomized material was compared with the X-ray fluorescence method (DELTA XRF, Olympus Corporation— Waltham, MA, USA). The images of the powder samples were taken with the Hitachi SU-3500 SEM (Tokyo, Japan). The flowability of the powder was measured if the volume of the powder was sufficient to run the Hall test; thus, only AISI 304 steel was measured. One of the most significant advantages of the proposed method is that we can perform experiments below this limit— the case of the Au-based alloy.

Ultrasonic spraying of solders and zinc alloys has been industrialized since the 1970s. Due to the thermal limitations of the ultrasonic tool materials, it was impossible to work above the melting point of aluminum alloys in the range of gigacycle fatigue. The other ultrasonic systems, mentioned in the literature, are mostly suitable to produce low-melting-point powders, such as Al-, Sn- or Pb-based alloys, or rely on consumable sonotrode. The novelty and advantage of our approach is that the system can produce any alloy, including the high temperature ones, without contamination [12,15,26].

**Figure 2:**
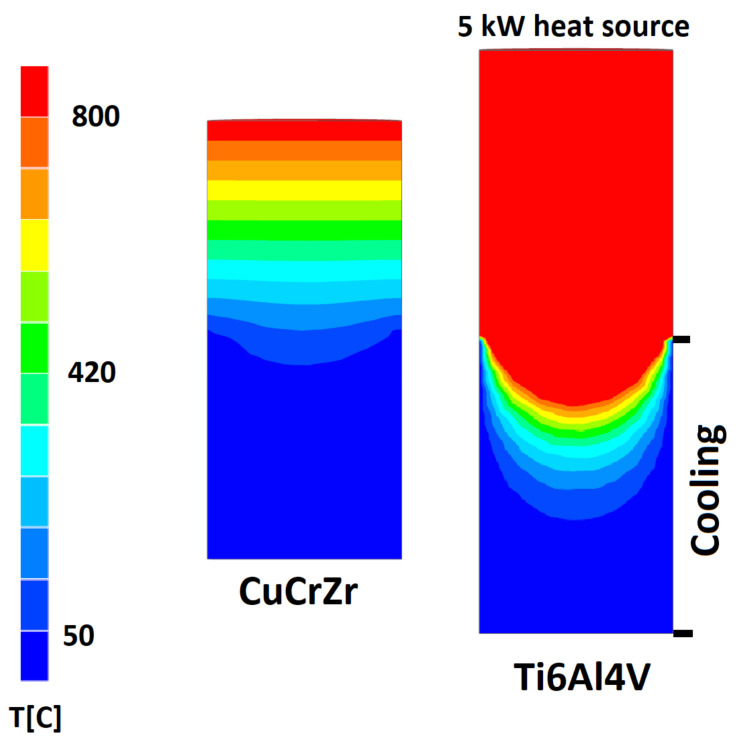
Temperature profile of 20 kHz Cu–Cr–Zr (Ampcoloy 972) and Ti–6Al–4V sonotrodes.

In the present work, this limitation was overcome by keeping the sonotrode cool as a constantly chilled sonotrode. System frequency drift was minimized as the arc heat input was balanced by the sonotrode heat flux. The high thermal conductivity (~300 W/m·K) of the Ampcoloy 972 ensures the sonotrode itself acts as a cold crucible. Using other materials with lower thermal conductivity (i.e. ~7 W/m·K for Ti–6Al–4V) and plasma source leads to the atomization of the sonotrode material itself. Three-dimensional temperature profiles of Ti–6Al–4V and Ampcoloy 972 sonotrodes were carried out with NX simulation (Siemens NX CAE build 1934, Simcenter Nastran solver, Munich, Germany). As it is shown in Figure 2, both 20 kHz longitude sonotrodes of Cu–Cr–Zr (Ampcoloy 972) and Ti–6Al–4V were loaded with constant 5 kW heat flux to demonstrate basics of cold-crucible approach. The temperature of Ti–6Al–4V overreached melting point of the alloy until nearly half of the sonotrode length, while Cu–Cr–Zr sonotrode temperature was maintained below melting point. The length of the sonotrodes is different due to various longitudinal wave velocities. Both sonotrodes are 38 mm in diameter and have 90 mm length for Ampcoloy and 120 mm for Ti–6Al–4V, which corresponds to 20 kHz resonant frequency at 297 K.

## 3. Results and Discussion

The sieve analysis of the atomized powders is shown in Table 1. Depending on the material, the total available yield for AM (particles less than 100 µm in diameter) reached 63.5% for the Au-based alloy. Such efficiency is comparable to laboratory-scale gas atomizers [6]. The coarsest particles were obtained in the case of the AMZ4 + W alloy; only 13.5% (11.8 + 1.7) were obtained below 100 μm, which is attributed to the increased viscosity of the tungsten alloyed melt [27]. The higher yield of unalloyed AMZ4 powder, 33.5% (23.3 + 10.2) below 100 µm, according to Pohlman et al. [15], could indicate the influence of the chemical composition on the surface tension of the melt. Such an indirect measurement of surface tension could be a useful and cost-effective tool for studying the effects of adding elements in various alloy systems.

Since all the tests were performed under the same conditions, optimized for AISI 304 steels, the process could be improved to increase the yield of AM grade particles. Further optimization can be performed using different sonotrode amplitudes and material overheating, although intense evaporation is expected with increased arc current. The most important parameter of ultrasonic atomization is the vibration frequency; the higher the frequency, the finer the particles [28].

Ultrasonic vibrations cause various effects on the liquid film, including capillary wave atomization and cavitation atomization [29]. Under selected conditions (300 A, 35 µm amplitude), certain test materials can be atomized with a changed mechanism explaining the major difference in particle size distribution.

The chemical composition of the ultrasonically atomized powders is shown in Table 2. Titanium Grade 2 and AISI 304 steel retained their standard composition with little contamination from the chamber. Minor chromium evaporation from 19.88% to 18.42% was observed in 304 AISI.

Intense fuming was observed during the Ni–Mn–Ga alloy atomization and the XRF results showed severe manganese depletion. The measured Mn content for particles with diameters was:

(1) 0 < 50 μm was 11.38 ± 0.2%;
(2) 50 < 100 μm was 13.40 ± 0.1%;
(3) 100 < 200 μm was 14.62 ± 0.1%;
(4) 200 < 500 μm was 31,48% ± 0.1%.

It shows that smaller particles tend to be depleted more in low manganese vapor pressure, either as a result of complete diffusion within particles or as a result of manganese boiling. As Smitll et al. [30] show, a depleted Mn layer up to 60 μm is probably the result of the solid-state diffusion and evaporation.

In the case of the AMZ4, slight zirconium depletion and iron contamination were observed. It might be related to the solid-state diffusion or evaporation and experimental setup changeover, respectively. As only minor quantities (less than 0.5 g for Au alloy) were atomized, the chemical composition of the atomized powder is sensitive to thorough cleaning of the chamber. The process of material changeover should be further developed.

In the case of the Au alloy, intense fuming was visible during the process. Considering the XRF results, it can be assumed that this was due to the intense evaporation of zinc. Low boiling point and high vapor pressure make zinc evaporate first during the arc melting of zinc-containing alloys [31].

Although the evaporation of zinc and manganese changes the initial chemical composition of the alloy, this effect can be considered beneficial when a reduction of trace elements is expected.

**Figure 3:**
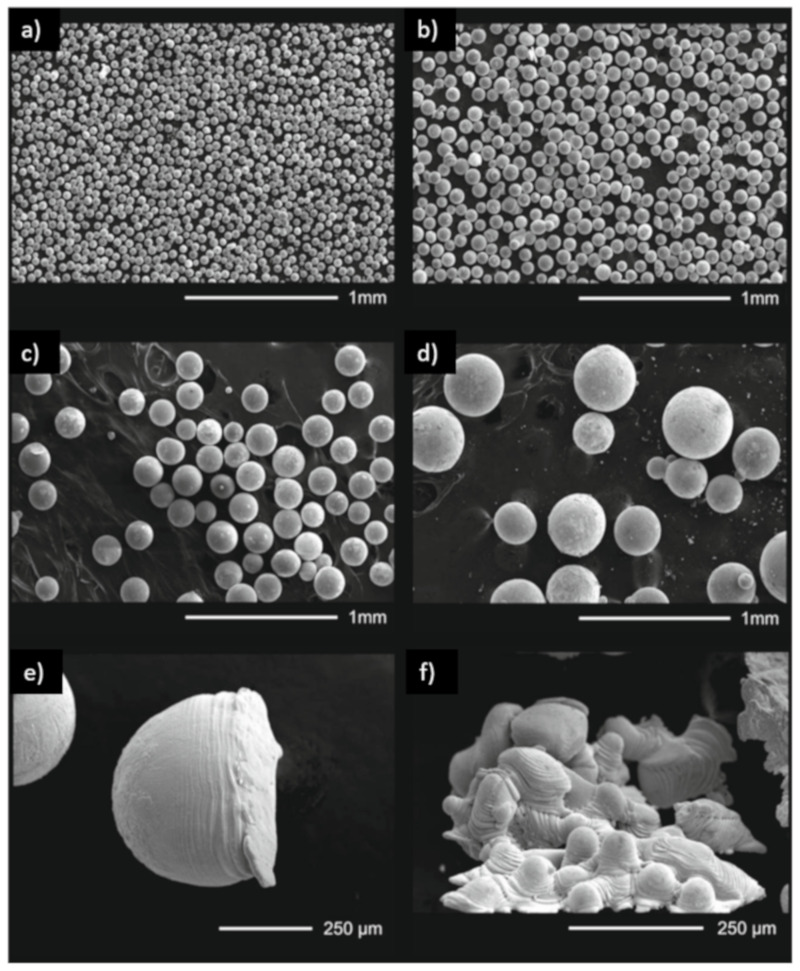
SEM images of (**a**) 0–50 µm (**b**) 50–100 µm (**c**) 100–200 µm (**d**) 200–500 µm of AISI 304 steel particles (**e**) the deformed powder particle and (**f**) the crystallized atomization site.

**Figure 4:**
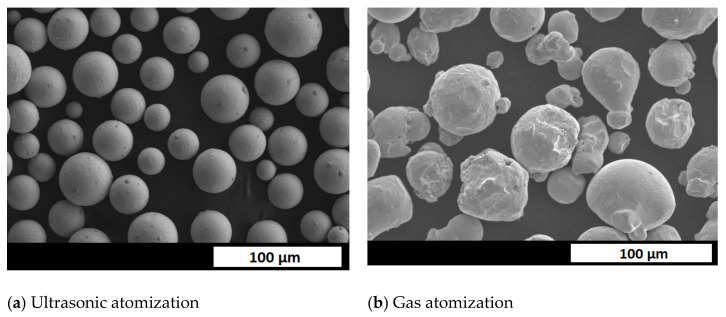
SEM images of <50 µm (**a**) ultrasonically atomized powder (**b**) gas-atomized powder.

SEM images of AISI 304 particles are shown in Figure 3. Regardless of the powder size, most of the particles are spherical in shape and contain several satellites. Such morphology is crucial for both the AM powder bed as well as cladding, providing both sufficient tap density and flowability. Depending on the process (e.g., hot pressing, laser cladding or LPBF), different grades of powder are preferred. The Hall test flowability results were below 12.1 ± 0.4 s for 50–100 µm and 10.2 ± 0.4 s for 100–200 µm. In the case of 304 steel, several defects were observed, as shown in Figure 3. Figure 3e shows a deformed particle, and Figure 3f shows a crystallized atomization site. One can see that large particles (~500 µm in diameter) were deformed as they collided with the chamber wall. The 316L ultrasonic atomized powder has been compared with standard commercially available materials with the same composition but prepared via the Inert Gas Atomization technique (MetcoAdd 316L-A, Oerlikon, Switzerland). Ultrasonically atomized fractions below <50 µm are shown in Figure 4a; in contrast, gas atomized powder is shown in Figure 4b. While ultrasonically atomized powder has almost uniform particle size, gas atomizer material has numerous fines in between particles with desired shape. Such distribution increased the tap density of powder, although it decreases flowability of particulate material (20 s Hall measurement [32]) making it less suitable for additive manufacturing. 

**Figure 5:**
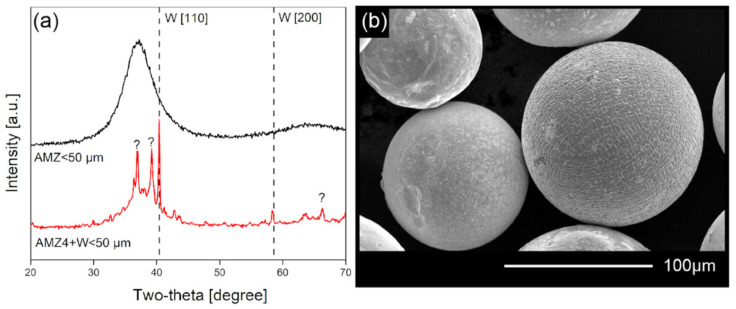
X-ray diffractogram for AMZ4 and AMZ4 + W < 50 μm particles (**a**) and the SEM image of 50–100 μm AMZ4 particles (**b**).

**Figure 6:**
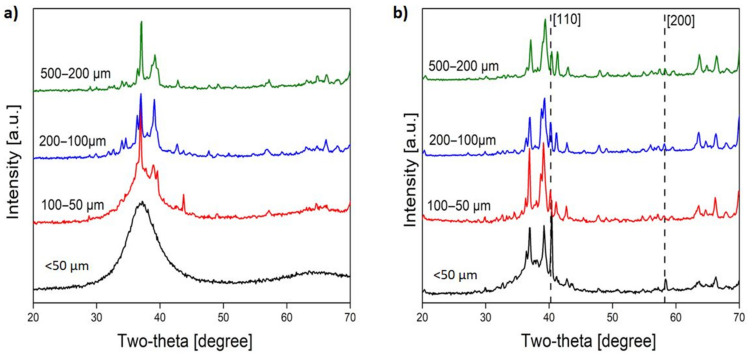
X-ray diffractogram for (**a**) AMZ4 and (**b**) AMZ4 + W.

The SEM image of AMZ4 grade 50–100 μm particles (Figure 5b) shows a well-defined dendritic structure which proves that the material tends to be partially crystalline beyond a certain particle diameter. No clear oxide layer was observed as the process chamber was purged with argon. The X-ray pattern (Figure 6) for AMZ4 alloyed with tungsten shows undissolved tungsten as well as unidentified crystalline phases. The AMZ4 and AMZ4+W form complex phase systems, including various ZrCu intermetallic and metastable phases. For us, the unimportant factor was if we could produce metallic glasses. The forms of crystalline phases, formed during the process, were of a secondary issue. The partial dissolution of the refractory metal in the melt is a well-known phenomenon used in composite production [33].

X-ray diffraction patterns for AMZ4 and AMZ4+W show that the finest AMZ4 particles <50 μm were amorphous. For both alloys, finer particles tend to have broader peaks, typically identified as smaller crystallites. Both the vitrification and grain refinement are attributed to the higher cooling rate in small particles [34].

**Figure 7:**
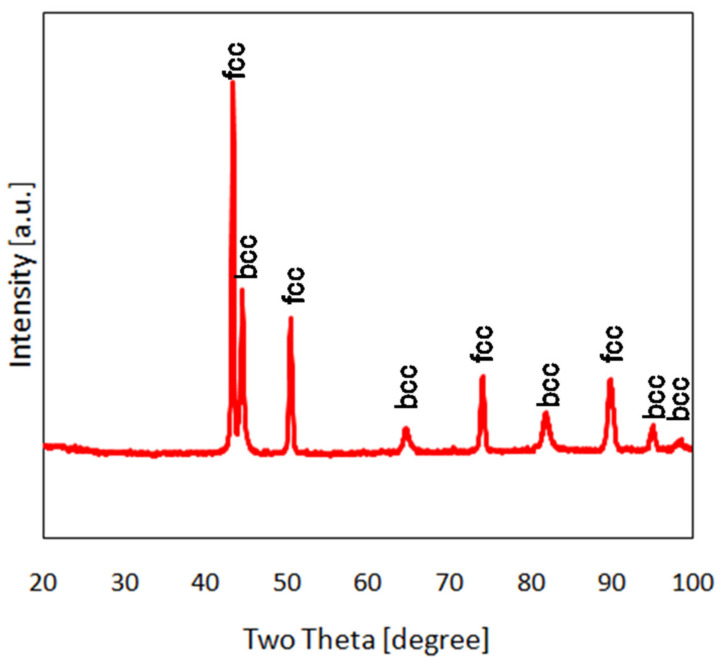
X-ray diffractogram for Fe–Cr–Mn–Ni alloy.

The X-ray diffraction pattern (Figure 7) obtained for the Fe–Cr–Mn–Ni alloy shows the coexistence of fcc and bcc phases. This is in line with previous experimental and theoretical studies for magnetic HEAs [25,29], which showed that such coexistence may be present for alloys with a valence electron concentration of 7.75, which refers to an equiatomic Fe–Cr–Mn–Ni alloy. Due to the multicomponent character of these alloys, a detailed analysis of the XRD patterns is not straightforward and therefore beyond the scope of this article.

As the cooling rate during atomization depends on several parameters, further optimization of the ultrasonic atomization of metallic glasses and crystalline alloys should be investigated. The heat flow during particle cooling is affected by radiation and convection, so increasing the pressure of the inert gas or using helium can increase the critical diameter of the amorphous particles and crystalline phase composition in other particles.

## 4. Conclusions

Production of six various alloys, via a novel cold crucible ultrasonic atomization technique with AM grade spherical powder up to 50%, have been presented:

(1) The powder particles tend to be spherical, regardless of the selected composition or particle size, with an exceptional Hall flowability of 10–12 s for AISI 304.
(2) The particle size distribution is dependent on the alloying elements, indicating the importance of surface tension in atomization mechanisms.
(3) Slight contamination and strong evaporation of manganese, zinc and zirconium were observed.
(4) The cooling rates achieved during the process were high enough to provide the vitrification of the AMZ4 Zr-based alloy. The tungsten alloying seems to decrease the glass-forming ability of this system.
(5) The proposed atomization technique was shown to be a useful tool for the development of new metallic materials for powder metallurgy and additive manufacturing. Moreover, it is possible to experiment with just a single or a few cubic centimeters of a particular material. This method is suitable for fabrication of any metallic material in small quantities, which is very convenient in experimental work.

## Figures and Tables

**Table 1:** Particle size distribution of atomized powders.

µm	Ti	304	AMZ4	AMZ4 + W	Ni–Mn–Ga	Fe–Cr–Mn–Ni	Au-Alloy
<50	5.8%	3.2%	10.2%	1.7%	2.3%	13.1%	21.1%
50–100	32.7%	41.9%	23.3%	11.8%	49.9%	42.1%	42.4%
100–200	46.2%	36.3%	20.9%	22.7%	38.0%	40.1%	22.3%
200–500	15.3%	18.6%	45.6%	63.8%	9.8%	4.7%	14.2%

**Table 2:** Chemical composition of atomized powders measured with XRF.

Material	Bulk (wt. %)	Powder (wt. %)
Ti Grade 2	Ti 99.84 ± 0.02 Fe 0.16 ± 0.02	Ti 99.53 ± 0.03 Fe 0.16 ± 0.02 Cu 0.15 ± 0.01 Ni 0.08 ± 0.01 Mn 0.08 ± 0.02
304 AISI	Fe 69.94 ± 0.13 Cr 19.89 ± 0.10 Ni 7.69 ± 0.09 Mn 1.48 ± 0.05 Cu 0.87 ± 0.03 Mo 0.13 ± 0.03	Fe 70.12 ± 0.12 Cr 18.42 ± 0.09 Ni 8.23 ± 0.09 Mn 1.63 ± 0.05 Cu 0.85 ± 0.03 Mo 0.16 ± 0.04 Si 0.59 ± 0.02
AMZ4	Zr 75.12 ± 0.18 Cu 22.94 ± 0.18 Nb 1.94 ± 0.05	Zr 73.81 ± 0.36 Cu 23.85 ± 0.34 Nb 1.94 ± 0.09 Fe 0.40 ± 0.16
AMZ4+W	Zr 56.83 ± 0.14 W 21.60 ± 0.14 Cu 20.29 ± 0.10 Nb 1.28 ± 0.02	Zr 55.67 ± 0.14 W 22.53 ± 0.15 Cu 20.49 ± 0.10 Nb 1.31 ± 0.02
Ni–Mn–Ga	Ni 33.98 ± 0.11 Mn 35.17 ± 0.12 Ga 30.85 ± 0.06	Ni 35.80 ± 0.12 Mn 31.80 ± 0.12 Ga 32.40 ± 0.06
Fe–Cr–Mn–Ni	Fe 28.68 ± 0.15 Cr 24.83 ± 0.14 Mn 24.97 ± 0.13 Ni 21.52 ± 0.14	Fe 28.52 ± 0.15 Cr 24.68 ± 0.13 Mn 21.74 ± 0.12 Ni 21.89 ± 0.13 Cu1.80 ± 0.05 W1.37 ± 0.05
Au-alloy	Au 63.20 ± 0.17 Cu 24.63 ± 0.14 Ag 9.75 ± 0.10 Zn 2.42 ± 0.05	Au 63.24 ± 0.10 Cu 27.61 ± 0.09 Ag 8.25 ± 0.05 Zn 0.90 ± 0.02

## Data Availability

The data presented in this study are available on request from the corresponding author. The data are not publicly available due to copyrights.
